# Isolated traumatic triceps tendon rupture following a motorcycle accident: a case report

**DOI:** 10.1093/jscr/rjae443

**Published:** 2024-07-06

**Authors:** Victor H Argueta, Daniela Saenz, Javier Ardebol

**Affiliations:** Department of Medical Research, Universidad Francisco Marroquín, 6ta calle final zona 10, Guatemala City 01010, Guatemala; Department of Medical Research, Universidad Francisco Marroquín, 6ta calle final zona 10, Guatemala City 01010, Guatemala; Department of Medical Research, Universidad Francisco Marroquín, 6ta calle final zona 10, Guatemala City 01010, Guatemala

**Keywords:** triceps, tendon, rupture, traumatic, isolated, injury

## Abstract

Triceps tendon ruptures are rare but significant injuries that impair upper extremity function. Despite their infrequency, recognizing this condition is crucial due to its severe impact on arm movement and strength. Patients typically present with posterior elbow pain, swelling, and bruising.This report details a complete triceps tendon rupture in a 34-year-old male following trauma. The patient exhibited classic symptoms: posterior elbow pain, significant swelling, and visible bruising, initially suggesting a severe soft tissue injury. Clinical examination and imaging confirmed a complete triceps tendon rupture. This case highlights the importance of considering triceps tendon rupture in patients with similar symptoms, particularly after trauma. Early recognition and accurate diagnosis are essential for timely surgical intervention, significantly improving functional recovery. Delayed diagnosis and treatment can lead to prolonged disability and poor outcomes, emphasizing the need for heightened awareness among healthcare providers regarding this rare but serious injury.

## Introduction

Triceps tendon ruptures are rare injuries that may lead to significantly compromised upper extremity function [1]. The triceps brachii muscle is responsible for upper extremity extension at the elbow joint; consisting of three separate heads, any of which might tear. Triceps tendon ruptures are less common than other tendon injuries. These ruptures commonly result after an eccentric muscle contraction, with a concomitant deceleration force applied against the actively contracting triceps muscle. Tears most frequently occur in the tendon insertion area compared to muscle–tendon junction or muscle belly [2, 3]. Most triceps injuries are traumatic in nature explaining why concomitant injuries are more common than isolated ones. In general, tricep tendon injuries are rare, being less common than biceps tendon ruptures or rotator cuff tears, which increases the risk of misdiagnosis and delayed treatment [1]. Understanding the mechanism of injury and clinical presentation of triceps tendon ruptures can help achieve a timely diagnosis as early management has shown better outcomes [1, 3]. Magnetic resonance imaging (MRI) is the gold standard imaging modality of choice. Despite modern advances in imaging quality and training, triceps tendon ruptures are challenging to diagnose using MRIs, especially when isolated [3].

This case report presents the clinical course, diagnosis, and management of an isolated complete triceps tendon rupture in a 34-year-old male patient following a motorcycle accident. Through this report, we aim to highlight the challenges associated with diagnosing and treating this injury, emphasizing the importance of prompt recognition and appropriate management to optimize patient outcomes and functional recovery.

## Case presentation

A 34-year-old male patient presents to the emergency department following a motorcycle accident. The patient reported pain on the lower aspect of his right arm, which limited mobility and function. Upon physical evaluation, a contusion was evident in the lower aspect of the right upper arm. The patient displayed pronounced swelling and discoloration in this region. Notably, he exhibited a visible deformity at the triceps muscle region of the upper arm, which was further supported with palpation.

**Figure 1:**
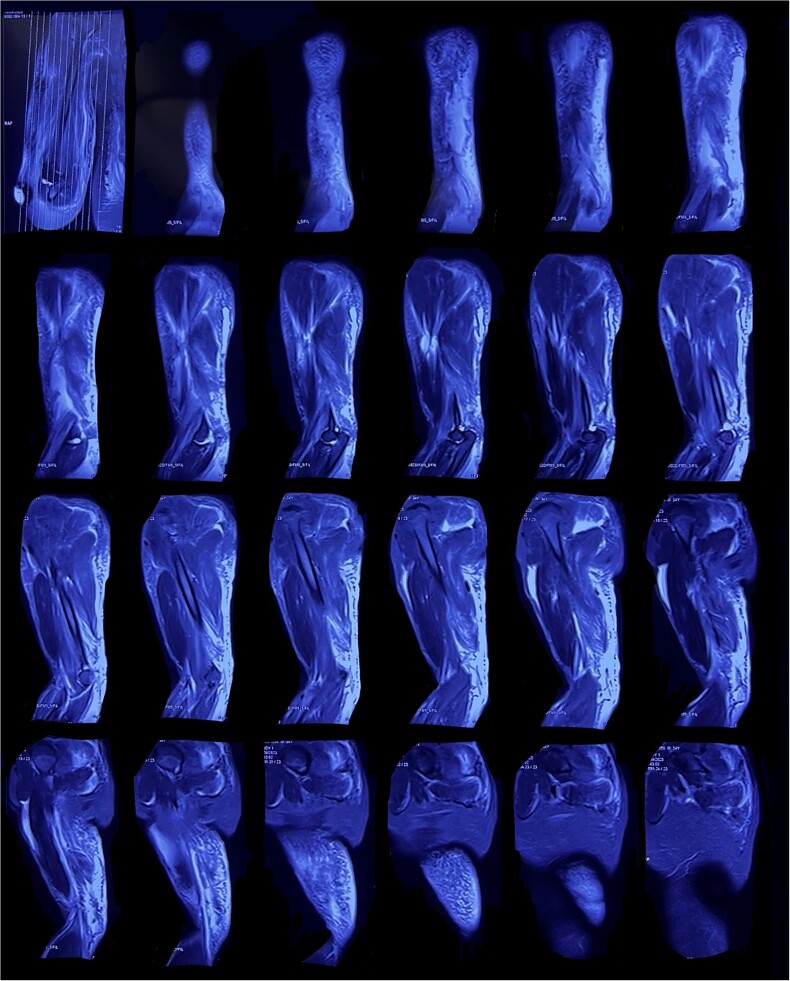
Left arm magnetic resonance imaging with evidence of a triceps tendon rupture.

To further assess the extent of the injury and confirm the initial clinical suspicion, imaging studies were promptly conducted. The MRI demonstrated hyperintense fibrillar patterns in the triceps muscles, indicative of traumatic contusion (Fig. 1). Additionally, the imaging disclosed the presence of significant soft tissue edema encompassing the mid and proximal thirds of the right upper arm. There was also significant synovial effusion within the elbow joint capsule, specifically in the lateral and external recesses, and a localized, dense, fluid collection within the posterior aspect of the right elbow joint measuring ~29 × 15 mm, indicative of a hematoma.

The anatomical correlation between the observed hematoma and the patient’s presenting symptoms, including pain, swelling, and deformity in the triceps region, suggested a complete rupture of the triceps tendon. These clinical findings reinforced the initial diagnosis of a complete triceps tendon rupture.

**Figure 2:**
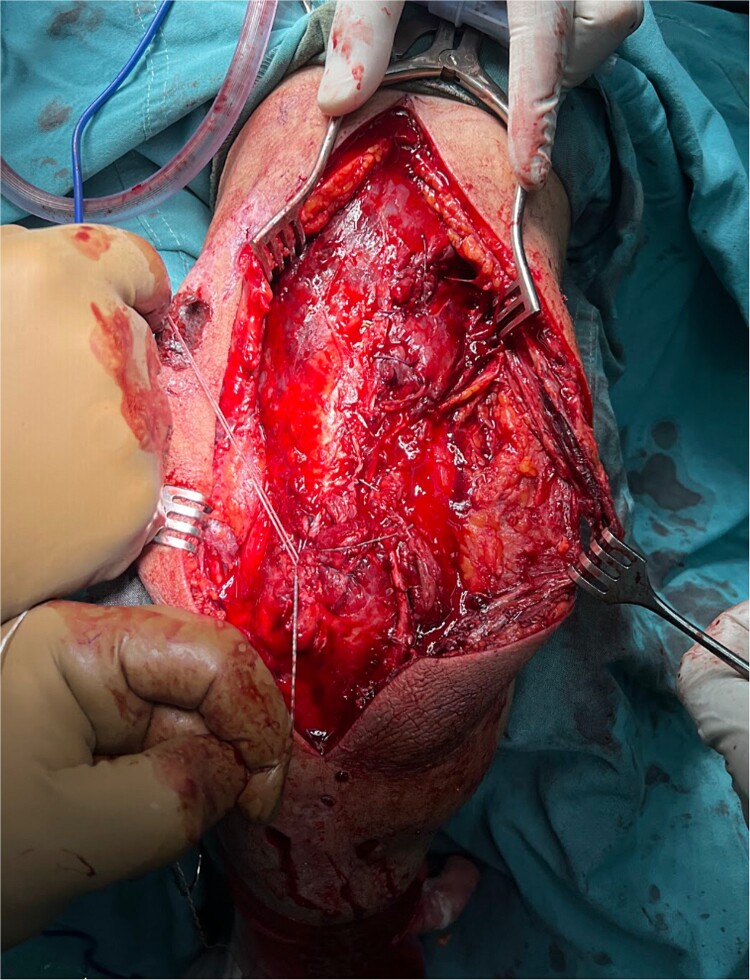
Left arm open triceps tendon repair with placement of modified Krackow suture technique and anchor suture on the olecranon tip.

**Figure 3:**
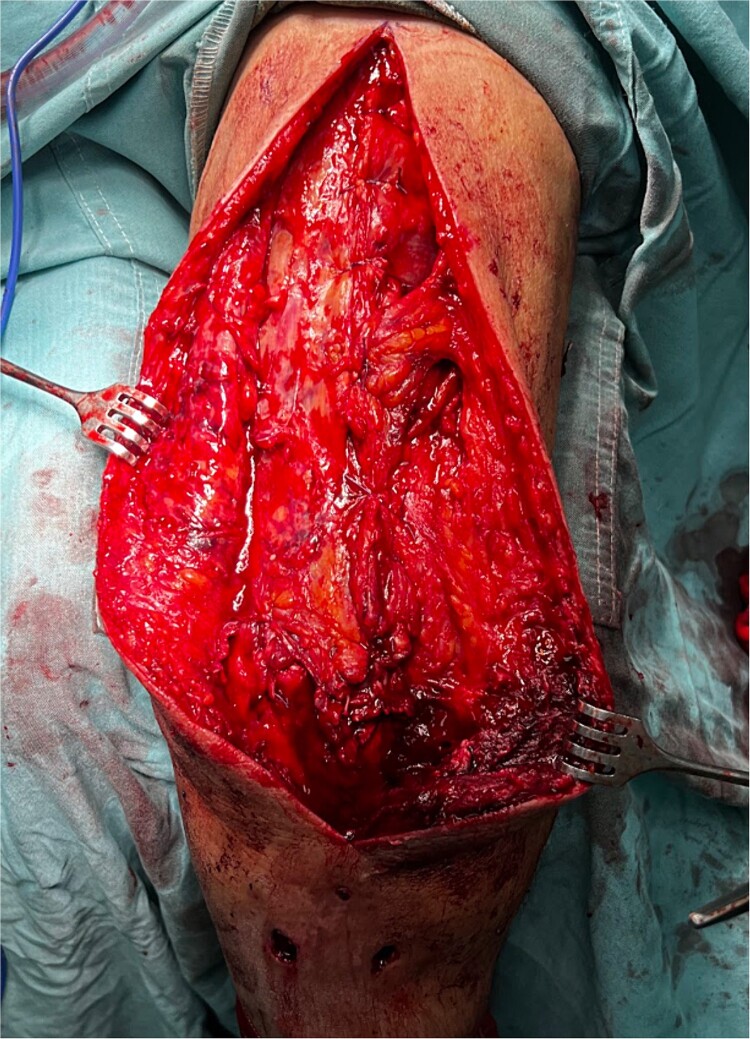
Left arm triceps tendon open surgical repair with a modified Krackow suture technique and anchor suture on the olecranon tip.

The patient underwent prompt triceps tendon repair. The surgical technique utilized a posterior approach to the humerus with dissection through fascial layers, and the identification and repair of the ruptured triceps tendon with a modified Krackow suture technique. Furthermore, a secure anchor (5.0 mm) (Figs 2 and 3) was placed on the olecranon tip. The medial, deep, and lateral triceps tendon heads were tagged with sutures and progressively tensioned towards the olecranon. Following tendon repair, the underlying fascia was reconstructed, followed by multilayered closure. The patient was discharged 1 week later, and was referred to physical therapy to continue the recovery process. After a 5 to 6 month recovery period, the patient recovered full range of motion and strength. Although ideal for assessing repair construct integrity, postoperative imaging could not be performed at the public hospital due to lack of resources.

## Discussion

Triceps tendon ruptures are uncommon injuries that significantly impair upper extremity function. These ruptures represent < 2% of all tendon injuries [4]. Sudden, forceful contractions of the triceps muscle, often during strenuous activities involving heavy lifting or forceful extension of the elbow have been associated with triceps tendon ruptures [5, 6].

Clinically, patients present with acute onset of pain, swelling, and weakness in the elbow region, often accompanied by a palpable defect near the olecranon process. Upon examination, a palpable defect may be noted in the tendon, along with weakness in elbow extension. Diagnosis is supported by physical examination, but confirmed with imaging such as ultrasound or MRI [6, 7]. Accurate diagnosis of triceps ruptures can be challenging, even with advanced imaging such as MRI. In this case, the MRI showed hyperintense fibrillar patterns in the affected muscles, soft tissue edema, and a synovial effusion within the elbow joint capsule. A localized hematoma within the posterior aspect of the right elbow joint provided compelling evidence of a complete triceps tendon rupture [8].

Management strategies vary depending on the extent of injury and patient characteristics. Conservative treatment, involving immobilization, physical therapy, and gradual return to activity, may be suitable for partial tears or patients with low functional demands [9]. In contrast, complete ruptures are best treated with surgical repair. Surgical techniques may vary, including direct repair with suture anchors or tendon graft augmentation in case of chronic or extensive injury [10].

Triceps tendon ruptures can significantly impact a person’s quality of life and upper extremity function [11]. Studies have demonstrated a 95% patient satisfaction rate after surgical treatment and 92% were able to return to preinjury function [3]. Early diagnosis and treatment are recommended as these may optimize results and minimize the risk of long-term complications. Complications of delayed treatment may vary depending on factors such as injury severity, patient comorbidities, and surgeon surgical experience [3, 11]. The most common complications are loss of function, muscle atrophy, chronic pain, joint stiffness, decreased strength, and potential development of arthritis in the affected joint. In cases of rerupture, revision surgery may be necessary [11]. Additionally, patients may experience prolonged recovery times and increased risk of infections. For managing stiffness, early but controlled physical therapy is essential, and if severe, manipulation under anesthesia or surgical release may be considered [3]. Patient education on activity modification and adherence to rehabilitation protocols is vital to prevent recurrence and ensure successful outcomes. Consistent follow-up care is crucial to monitor progress and address any complications promptly.

## Conclusions

This case report describes a rare isolated traumatic triceps tendon rupture. This injury benefits from a prompt diagnosis and repair to achieve optimal results. The aim of a surgical repair is to restore the triceps tendon function which may translate to improved quality of life. It is recommended to have a multidisciplinary approach pre- and postoperatively to optimize results.
